# Gut Microbiota and Neurovascular Patterns in Amnestic Mild Cognitive Impairment

**DOI:** 10.3390/brainsci15060538

**Published:** 2025-05-22

**Authors:** Alexis B. Kazen, Laura Glass Umfleet, Fatima A. Aboulalazm, Alexander D. Cohen, Scott Terhune, Lilly Mason, Shawn Obarski, Malgorzata Franczak, Tammy Lyn Kindel, Yang Wang, John R. Kirby

**Affiliations:** 1 Department of Microbiology and Immunology, Medical College of Wisconsin, Milwaukee, WI 53226, USA; akazen@mcw.edu (A.B.K.); faboulalazm@mcw.edu (F.A.A.); sterhune@mcw.edu (S.T.); jkirby@mcw.edu (J.R.K.); 2 Department of Neurology, Medical College of Wisconsin, Milwaukee, WI 53226, USA; lillymason525@gmail.com (L.M.); sobarski@mcw.edu (S.O.); mfranczak@mcw.edu (M.F.); yangwang@mcw.edu (Y.W.); 3 Department of Radiology, Medical College of Wisconsin, Milwaukee, WI 53226, USA; acohen@mcw.edu; 4 Department of Surgery, Medical College of Wisconsin, Milwaukee, WI 53226, USA; tkindel@mcw.edu; 5 Department of Biophysics, Medical College of Wisconsin, Milwaukee, WI 53226, USA

**Keywords:** microbiome, virome, Alzheimer’s disease, shotgun metagenomics, neurovascular dysfunction, neurodegeneration, dementia, blood–brain barrier

## Abstract

**Background/Objectives:** The interplay between the gut microbiome (GMB) and neurovascular function in neurodegeneration is unclear. The goal of this proof-of-concept, cross-sectional study is to identify relationships between the GMB, neurovascular functioning, and cognition in amnestic mild cognitive impairment (aMCI), the prototypical prodromal symptomatic stage of Alzheimer’s disease (AD). **Methods:** Participants (*n* = 14 aMCI and 10 controls) provided fecal samples for GMB sequencing (16S and shotgun metagenomics), underwent MRI, and completed cognitive testing. Cerebral vascular reactivity (CVR), cerebral blood flow (CBF), and arterial transit time (ATT) were assessed. Statistical analyses evaluated the relationships between discriminatory taxa, cerebrovascular metrics, and cognition. **Results:** Sequencing revealed differentially abundant bacterial and viral taxa distinguishing aMCI from controls. Spearman correlations revealed that bacteria known to induce inflammation were negatively associated with CVR, CBF, and cognition, and positively associated with ATT. A reciprocal pattern emerged for the association of taxa with gut health. **Conclusions:** Our results provide preliminary evidence that pro-inflammatory gut bacterial and viral taxa are associated with neurovascular dysfunction and cognitive impairment in prodromal AD, highlighting their potential as candidate microbial biomarkers and targets for early intervention.

## 1. Introduction

Alzheimer’s disease (AD) is the most prevalent neurodegenerative disease in older adults, and is a growing epidemic worldwide. The AD continuum includes preclinical AD (asymptomatic individuals with biomarker evidence of pathology), prodromal AD or mild cognitive impairment (MCI; mild but measurable cognitive decline, often amnestic, without functional impairment), and AD dementia (marked cognitive and functional decline) [1,2,3]. Despite decades of research, there remains no effective treatment for AD. Treatment failures can be attributed, at least in part, to an incomplete understanding of the pathogenic mechanisms of AD, which underscores the urgent need to examine alternative pathogenic mechanisms and therapeutic targets for AD.

Within the last decade, the gut microbiome (GMB) has been increasingly implicated in the progression of AD pathogenesis, even at the preclinical and prodromal stages of the disease [4,5]. Pathways linking the gut and brain in AD have been proposed [6]. GMB studies (mostly using 16S rRNA sequencing) have revealed bacterial taxa capable of distinguishing dementia due to AD from prodromal AD and controls, along with associations with cerebral spinal fluid (CSF) biomarkers of AD [4,7,8] and brain MRI in MCI and AD [9,10]. Of the few studies that have examined the relationship between the GMB and brain MRI in AD, there is only one known study examining the associations between the GMB and functional MRI in AD [9], and one study examining diffusion MRI in AD [10]. Additionally, only one other study has investigated gut virome differences in prodromal AD [11]. To our knowledge, no study has examined the interplay between bacterial and viral taxa and neurovascular changes in AD.

Considering the substantial evidence connecting early vascular contributions to AD pathophysiology and dementia, cerebrovascular dysfunction has emerged as a major contributor to cognitive decline and disease progression in AD [12]. The blood–brain barrier (BBB), a neurovascular structure responsible for regulating the passage of cells and molecules to and from the central nervous system (CNS), has been identified as a key player in this process [13]. Disruption of the BBB allows the influx of neurotoxic blood-derived molecules, cells, and microbial pathogens into the brain, triggering inflammatory and immune responses that can initiate various pathways of neurodegeneration [14,15]. Growing evidence suggests that dysfunction and/or breakdown of the BBB, as well as a reduction in and/or dysregulation of cerebral blood flow (CBF as measured by MRI), may occur in sporadic AD and experimental models of the disease prior to cognitive decline, Aβ deposition, and brain atrophy [15,16]. The cerebral vasculature is the locus where multiple pathogenic processes converge and contribute to cognitive impairment [17], and has given rise to speculation regarding the existence of a common pathological triad model that consists of vascular damage, neurodegeneration, and neuroinflammation [18], expanding upon the traditional understanding of AD. Recent neuropathological research has provided evidence to support this notion, demonstrating that systemic infection can alter brain cytokine levels and exacerbate cerebral hypoperfusion and BBB leakiness associated with AD, independently of the level of insoluble Aβ [16]. Mouse models have demonstrated that GMB disruption can trigger neurovascular dysfunction related to AD risk [19], suggesting that microbial pathogens may influence AD pathogenesis through chronic inflammation to disrupt the neurovascular unit [20], alter metabolite production to affect vascular function [21,22], or affect CBF regulation directly [23]. The complex crosstalk between neuroinflammation, immune responses, and vascular changes in the context of neurodegeneration remains poorly understood. Thus, it is essential to acquire a deeper understanding of the underlying pathophysiology. Mounting evidence indicates the involvement of inflammation-inducing gut bacterial taxa in the onset and progression of AD, and provides insights into the possible microbial mechanisms involved in the onset and progression of AD.

The purpose of the current study is to understand the interplay between gut microbes, neurovascular dysfunction, and cognition in patients clinically diagnosed with amnestic MCI (aMCI) believed to be caused by AD compared to cognitively unimpaired older-adult controls. We examined 16S and shotgun metagenomics sequencing, multimodal neuroimaging metrics to assess neurovascular dysfunction, and performance in neuropsychological measures of memory, language, processing speed, and executive functioning.

## 2. Materials and Methods

### 2.1. Participants

All subjects provided written informed consent prior to participation in this study, which was approved by the local Institutional Review Board and conducted in accordance with the Declaration of Helsinki and its later amendments or comparable ethical standards. Participants (*n* = 24; 63 to 84 years old; 58% female, 96% White) were recruited from our ongoing NIA R21 study, which is a cross-sectional, non-interventional study that examines associations between bacterial taxa (stool), neurovascular functioning (brain MRI), and cognition. We selected these 24 participants for the current study because both their stool and neuroimaging data had been processed and analyzed. Participants with aMCI/prodromal AD (*n* = 14) were diagnosed with this condition by a clinical neuropsychologist or memory disorder neurologist as part of routine clinical care at Froedtert and the Medical College of Wisconsin. The MCI diagnosis was documented in a recent clinical visit with a neurologist or neuropsychologist within 6 months or less of the first study visit. Ten participants were cognitively unimpaired control subjects, as supported by a brief neuropsychological research evaluation. Exclusion criteria for all participants included a history of moderate-to-severe traumatic brain injury, a brain tumor, symptomatic stroke, severe psychiatric illness (i.e., schizophrenia and bipolar disorders), intellectual disability, dementia/a major neurocognitive disorder, end-stage renal or liver disease, drug addiction, alcoholism, inflammatory bowel disease, a recent history of viral infections, antibiotic use within two months before stool sample collection, and an age younger than 60 years. Participants with a history of hypertension (aMCI = 25%; controls = 40%), diabetes (aMCI = 14%; controls = 0%), or high cholesterol (aMCI = 43%; controls = 20%) were able to participate in the current study, as these are common health conditions in older adult populations and AD risk factors. We acknowledge that our participants are not diverse in race, but are diverse in terms of other sample characteristics, such as medical history and gender. Using non-probability sampling methods, we approached all potentially eligible individuals based on our inclusion and exclusion criteria noted above.

In this proof-of-concept, cross-sectional study, each participant completed two study visits. The first visit included collection of demographics, medical and family history information, and blood samples. Participants were sent home with a fecal collection kit, provided by our institution’s Center for Microbiome Research (CMR). Patients underwent MRI at the second study visit. Sample characteristics, including demographics (age, education, sex), BMI, and parental history of dementia, are reported in Table 1. MCI and control groups did not statistically significantly differ in these variables.

### 2.2. Data Collection

#### 2.2.1. Stool

Participants were provided with in-home fecal collection kits with instructions during their first study visit. They completed a 7-day diet log prior to providing and shipping a stool sample according to standard instructions provided by the CMR. Samples were frozen at −80 °C until processed by the CMR. Genomic DNA was extracted using the Qiagen PowerLyzer PowerSoil Kit (QIAGEN Sciences, Germantown, MD, USA). Purified genomic DNA was then submitted to the University of Wisconsin–Madison Biotechnology Center for 16S and shotgun metagenomics sequencing.

16S rDNA bacterial gene sequencing: 16S rDNA libraries were generated using the V3-V4 region spanning primers 341F to 806R at the University of Wisconsin–Madison Biotechnology Center using the Illumina TruSeq DNA library prep system. PCR products were sequenced on the Illumina MiSeq platform using the 2 × 300 bp protocol. Amplicons were generated using a dual-indexing amplification strategy and optimized for sequencing.

Whole-metagenome sequencing for human samples: Sequences were generated on the Illumina NovaSeq X Plus platform, producing 2 × 150 paired-end reads on an S4-flow cell, at the University of Wisconsin–Madison Biotechnology Center. The depth of coverage was ~50 M reads per human sample.

#### 2.2.2. Magnetic Resonance Imaging (MRI)

MRI Acquisition. All imaging was performed on the MCW research-dedicated GE Signa Premier 3T scanner using the Nova Medical 32-channel phased-array head coil. Anatomical imaging included a high-resolution 3D T1-weighted MPRAGE (voxel size = 0.47 × 0.47 × 0.5 mm). Functional imaging utilized an advanced multiband multi-echo (MBME) sequence (TR = 1000 ms, three echoes at TE = 11, 30, 49 ms) with multiband factor = 4. The complete MRI protocol parameters have been previously described in detail [24,25,26]. Cerebrovascular reactivity was assessed using our established breath-holding (BH) paradigm, consisting of four cycles of 16 s breath-holds alternating with recovery periods. For cerebral blood flow quantification, we employed Hadamard-encoded, multiple-delay 3dPCASL with seven post-labeling delays (1.0–3.7 s) and ATT correction using signal-weighted delay methodology [27,28].

All neuroimaging data underwent rigorous quality assessment. Images were visually inspected for expected gray/white matter contrast, minimal negative values, and artifact presence. Respiratory compliance was monitored during BH scans, and only subjects demonstrating four clear BH periods were included. One MCI subject did not undergo BH scanning, one was excluded due to poor BH scan quality, and another was excluded due to inadequate CBF scan quality. See Table 2 for final sample sizes for the CVR, CBF, and ATT analyses.

#### 2.2.3. Clinical Assessments

Controls: Controls completed a medical history questionnaire and measures of verbal memory (Rey Auditory Verbal Learning Test, RAVLT) [29], psychomotor processing speed and mental flexibility (Trail-Making Test, Parts A and B) [30], and language (letter and category fluency) [30]. To confirm the absence of dementia-related behavioral changes in these participants, a study partner completed the Quick Dementia Rating System [31]; all scores fell within the normal range of <1.5 out of 30.

aMCI Group: Clinical neuropsychological data on participants diagnosed with aMCI were obtained via retrospective electronic medical record review. These data include demographic variables, MCI diagnosis, medical history and medications, psychiatric history, and neuropsychological test scores. Common cognitive testing data elements were identified from clinical neuropsychological exams and entered into the study database. Common data elements were the Trail-Making Test (TMT, Parts A and B) [30], letter and category fluency tasks (composite score was created from one of three different versions [30,32,33]), and verbal-memory spontaneous delayed recall (composite score was created from Hopkins Verbal Learning Test—Revised [34] or RAVLT [29]). Two patients had a recent cognitive screening assessment as part of their visit with a memory disorder neurologist, but did not have recent comprehensive neuropsychological testing. One patient had neuropsychological testing but no TMT results. Therefore, the sample size for common cognitive testing data elements for the aMCI group ranged from 11 to 12.

### 2.3. Data Analysis

#### 2.3.1. Gut Microbiome

16S sequencing and analysis: 16S V3-V4 amplicon libraries were generated at the University of Wisconsin–Madison Biotechnology Center. PCR products were sequenced on the Illumina MiSeq platform using the 2 × 300 bp protocol. Paired-end reads were analyzed using Quantitative Insights into Microbial Ecology (QIIME2), which is updated regularly [35] and was processed by us using standard workflows, as described previously [36]. Taxonomy was assigned to ASVs against the SILVA 138 reference database. Changes in the abundance of individual taxa were also analyzed using traditional univariate statistical methods. Linear discriminate analysis effect size (LEfSe) [37] was used to determine discriminant ASVs (bacterial taxa) and confirmed via Random Forest machine learning analyses and MaAsLin2 v. 1.16.0 using RStudio (version 2023.12.1+402 and R version 4.3.3) [38,39]. Correlation statistical analyses were performed using the “corr.test” function of the psych R package to calculate Spearman’s rank correlation coefficients and *p*-values [40]. The ggcorrplot R package, using ggplot2, was utilized to visualize correlation matrices as circles of neurovascular patterns against differentially abundant taxa [41,42]. Plots were arranged together using the patchwork R package v. 1.3.0 [43].

Whole-genome shotgun sequencing and analysis: Sequences were generated on the Illumina NovaSeq platform, producing 2 × 150 paired-end reads on an S4-flow cell, at the University of Wisconsin–Madison Biotechnology Center. Raw sequences were quality-filtered, assembled to generate contigs, and processed to create metagenomes (MAGs). We utilized anvi’o for all metagenomics processing and visualization, as described previously [44]. MAGs were manually curated based on a threshold of 50% completion (relative to a reference) or a genome size greater than 2 Mb and less than 10% redundancy based on bacterial single-copy-core gene collection [45]. RStudio was used to run Random Forest (rf) or MaAsLin2 [38,39] packages to identify the top discriminatory MAGs between samples. LEfSe [37] was also used to determine discriminant MAGs. Spearman’s Rho correlational analysis, as described above for the 16S analyses, was conducted to confirm associations between bacterial taxa and clinical data. Comparisons between *Turicibacter* and *Bilophila* were conducted using GraphPad Prism v10. Equal abundance subset analysis of *Bilophila wadsworthia* was conducted by choosing samples whose abundances were approximately the same value (*p* > 0.999 via Mann–Whitney U test comparing control (*n* = 5) and aMCI (*n* = 7)). The gene coverages from this subset of samples were run through LEfSe to identify differentially abundant genes between *Bilophila* associated with controls vs. *Bilophila* associated with aMCI.

Whole-genome shotgun virome analysis: The contigs file generated after quality controlling and assembling the raw sequencing reads, as described above, was used for downstream virome analyses. Viral contigs were identified using Virsorter2 [46] and were quality controlled using CheckV [47]. Viral genes, including auxiliary metabolic genes (AMGs) were functionally annotated using DRAMv [48]. Viral contigs were manually curated as previously described [49] to achieve greater confidence in viral identification. As with the bacterial MAGs, high-quality viral contigs that were present in at least three samples underwent discriminant analysis using LEfSe, Random Forest, and MaAsLin2. Viral contigs underwent Spearman’s Rho correlational analysis as described above to further identify and confirm associations with the cognitive and neurovascular data. Virus hosts were identified using VirHostMatcher-Net [50], or via the NCBI blastn suite if a contig was unable to be identified using VirHostMatcher-Net. The lytic vs. lysogenic state of the viral contigs was determined by running the BAM files (described above) through VIBRANT [51] and running PropagAte [52] on the VIBRANT-predicted viral contigs to determine the viral-to-bacterial ratio (VBR). As with the 16S and bacterial MAGs, Spearman’s Rho correlational analysis was run comparing the viral contigs with clinical and neurovascular measures of cognitive impairment. Visualization of these correlations was generated in the same way as described for 16S and bacterial MAGs.

#### 2.3.2. Neuroimaging

MRI data were analyzed using the AFNI [53], FSL [54], and Advanced Normalization Tools (ANTS) programs [55]. Functional data preprocessing followed established neuroimaging procedures [26,56] to be adapted for our multi-echo acquisition. Anatomical images were co-registered to MNI space through linear and nonlinear transformations. The functional data processing included volume registration, multi-echo independent component analysis (ME-ICA [57,58]) implementation via tedana v 23.0.1, spatial normalization, and 6 mm FWHM Gaussian smoothing. Additional methodological details are available in our previous publication [24]. Cerebrovascular reactivity was quantified using phys2cvr version 0.18.6, which generated time-shifted regressors for optimal CVR estimation. The analysis employed a breath-hold regressor derived from convolution of a square wave with the respiration response function [59], time-shifted from −9 to 9 s in 1 s increments. Perfusion data processing involved registering the M0 reference image to the anatomical scan using epi_reg [54], followed by transformation of CBF and ATT maps to standard space using the computed transformation matrices.

Statistical analyses included voxelwise comparisons between aMCI and control participants using 3dttest++ with age and sex covariates. The results were thresholded at *p* < 0.01 and cluster-corrected using 3dClustSim [60] at α < 0.05. Mean values were extracted from significant clusters and compared between groups using Mann–Whitney tests. Spearman correlations examined relationships between imaging metrics, cognitive performance, and bacterial taxa. Given the proof-of-concept nature of this investigation, both significant (*p* ≤ 0.05) and trending (*p* < 0.10) results are reported.

## 3. Results

### 3.1. Cognitive Measures and Neurovascular Data for aMCI and Control Cohorts

**Figure 1:**
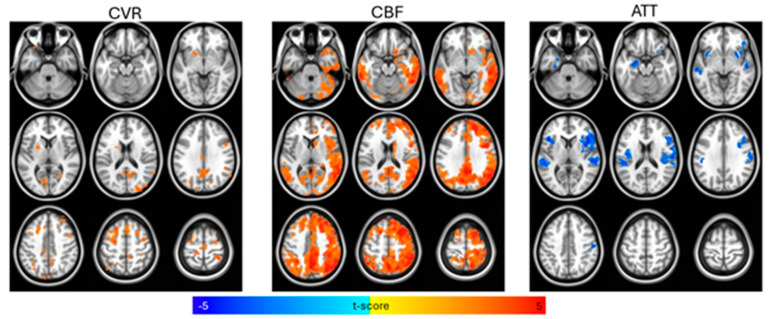
Voxelwise *t*-test comparisons between control and amnestic Mild Cognitive Impairment (aMCI) groups for cerebral vascular reactivity (CVR), cerebral blood flow (CBF), and arterial transit time (ATT), controlling for age, biological sex, and gray matter density, with statistical significance set at *p* < 0.01 (cluster-size correction at α < 0.05). CBF and CVR were higher and ATT was lower for the control group compared to the aMCI group.

Reported in Table 2 are means, *SD*s, and parametric (independent-sample *t*-tests) and non-parametric (Mann–Whitney test) group comparison (control vs. aMCI) results for cognitive measures, as well as CVR, CBF, and ATT values. The aMCI group had lower mean scores on tests of episodic memory (delayed recall), psychomotor processing speed (Trails A), executive functioning (Trails B), and language (category fluency, not letter fluency) (all *p* ≤ 0.01). Subsequent analyses with cognitive measures excluded letter fluency to focus on clinical measures that differentiated the aMCI group from the control group. Illustrated in Figure 1 are the results of the independent-sample *t*-tests for control–aMCI comparisons with age, biological sex, and GM density as covariates for CVR, CBF, and ATT. The control group had higher CVR and CBF and lower ATT values compared to aMCI participants at the cluster-wise corrected *p*-value threshold of 0.01. This was also evident with Mann–Whitney non-parametric group comparisons—the CVR (*U* = 17.00, *z* = −2.84, *p* = 0.003) and CBF (*U* = 24.00, *z* = −2.54, *p* = 0.010) values were higher in controls, whereas ATT values were lower in controls (*U* = 105.00, *z* = 2.48, *p* = 0.012). Significant and trend-level correlations emerged between cognitive measures and neurovascular metrics. Most notably, higher executive functioning (TMT-B) scores were positively correlated with CVR and CBF (*r_s_* = 0.527, *p* = 0.020 and *r_s_* = 0.417, *p* = 0.067, respectively). A negative association between TMT-B and ATT (*r_s_* = −0.357; *p* = 0.122) was observed, but did not reach the *p* < 0.10 threshold for trending significance. Similar patterns emerged with other cognitive measures, mainly delayed recall and category fluency scores (Table 3).

### 3.2. 16S Sequencing Analysis of aMCI and Control Participants

**Figure 2:**
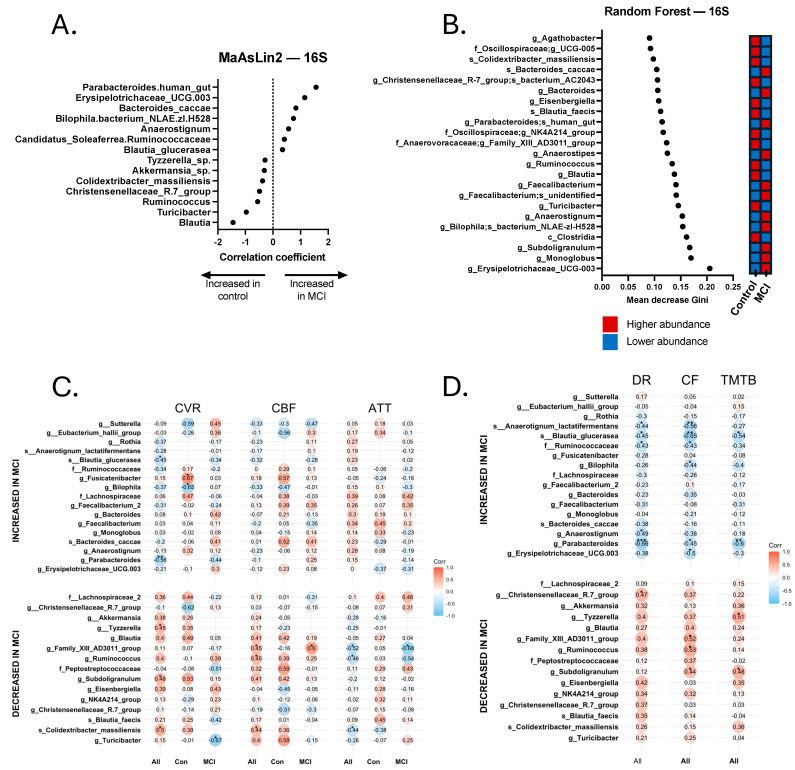
Discriminatory taxa from the 16S Random Forest and MaAsLin2 analyses and correlations with neurovascular and cognitive data. (**A**) Discriminatory taxa identified via MaAsLin2 using a *p*-value cutoff of 0.05. (**B**) Discriminatory taxa identified via Random Forest using a Gini score cutoff of 0.09. Discriminatory taxa identified by Random Forest and MaAsLin2 underwent Spearman’s Rho correlational analysis to identify taxa that were significantly correlated with neurovascular metrics, including cerebrovascular reactivity (CVR), cerebral blood flow (CBF), and arterial transit time (ATT). Correlations were calculated separately for all 24 participants (All), 14 aMCI participants (MCI), and 10 controls (Con). Discriminatory taxa also underwent Spearman’s Rho correlational analysis for cognitive analyses, including delayed recall (DR), category fluency (CF), and Trail-Making Test B (TMTB). Correlations for cognitive tests were calculated for the total sample (All). (**C**) Spearman correlations for cerebrovascular analyses. (**D**) Spearman correlations for cognitive tests. * *p* < 0.05, ** *p* < 0.01, *** *p* < 0.001.

DNA from stool samples from both the aMCI and control cohorts was assessed for changes in GMB. The 16S sequencing yielded 269 ASVs across both cohorts. None of the tested measures of alpha and beta diversity were significantly different between the two groups (*p* > 0.05). However, we did identify several differentially abundant bacterial taxa in each cohort (Figure 2A,B). Several ASVs associated with aMCI participants are members of bacterial species known to be pro-inflammatory or have previously been associated with cognitive impairment, such as *Bilophila* (identified via all methods of differential abundance) and *Faecalibacterium* (identified via Random Forest analysis). Bacteria associated with a healthy GMB, including *Akkermansia* (identified via MaAsLin2 analysis) and *Turicibacter* (identified via Random Forest and MaAsLin2 analysis), were more abundant in controls (Figure 2A,B).

Relationship between Bacterial Taxa, Neurovascular Patterns, and Cognition. Across all participants with both GMB and neurovascular data, we found a pattern of positive and negative Spearman correlations (significant and non-significant or trend-level). Pro-inflammatory bacterial taxa that were more abundant in the aMCI group, such as *Bilophila*, were generally negatively correlated with CVR and CBF values and positively correlated with ATT. Conversely, bacterial taxa associated with a healthy gut, such as *Akkermansia*, *Turicibacter*, and *Subdoligranulum*, were more abundant in controls, and were typically positively correlated with CVR and CBF and negatively correlated with ATT. Statistically significant and trend-level findings between 16S and neurovascular metrics for the total sample, controls, and MCI are displayed in Figure 2C,D. Notably, not all values reached the *p* < 0.10 threshold, but those that did not are still displayed for visual reference.

Similarly, we also found a pattern of positive and negative Spearman correlations across all participants with both GMB and cognitive data (Figure 2C,D). Pro-inflammatory bacteria, such as *Bilophila*, *Erysipelotrichaceae UCG-003*, and *Anaerostignum*, were associated with worse cognitive functioning, whereas bacteria associated with a healthy gut, including two *Christensenellaceae R-7* group bacteria, *Subdoligranulum*, and *Eisenbergiella*, were associated with better cognitive functioning. Significant and trend-level correlational findings emerged more frequently with tests of delayed recall (memory) and category fluency (language). For 16S results, 9 of the 17 bacterial taxa that were associated with cognitive functioning (significant to trend-level) also showed significant or trend-level significant correlations with neurovascular metrics. For instance, *Subdoligranulum g.* was significantly positively correlated with TMT-B (*r_s_* = 0.476, *p* = 0.029) and category fluency (*r_s_* = 0.443, *p* = 0.039), as well as CVR (*r_s_* = 0.483, *p* = 0.023) and CBF (*r_s_* = 0.408, *p* = 0.053). Although not statistically significant, the correlation coefficient between *Subdoligranulum g.* and ATT was a negative value (*r_s_* = −0.197, *p* = 0.367). *Eubacterium g.* was positively correlated with delayed recall (*r_s_* = 0.401, *p* = 0.065), category fluency (*r* = 0.519, *p* = 0.013), and CBF (*r_s_* = 0.447, *p* = 0.033), and was negatively correlated with ATT (*r_s_* = −0.524, *p* = 0.010). *Tyzzerella sp*., which positively correlated with all cognitive measures (trend-level-to-significant correlational values), also positively correlated with CVR (*r_s_* = 0.451, *p* = 0.035) and CBF (*r_s_* = 0.409, *p* = 0.053), and a negative correlational value emerged with ATT (*r_s_* = −0.248, *p* = 0.254), but did not meet the threshold for trending significance. Human gut-associated *Parabacteroides* was significantly negatively correlated with all cognitive measures (*p*s < 0.05), as well as CVR (*r_s_* = −0.562, *p* = 0.006). *Blautia glucerasea* was significantly negatively correlated with all cognitive measures (*p*s < 0.05), along with CVR (*r_s_* = −0.451, *p* = 0.035) and CBF (*r_s_* = −0.321, *p* = 0.135), and positively correlated with ATT (*r_s_* = 0.227, *p* = 0.299), although the latter two values did not reach the threshold for trending significance.

### 3.3. Shotgun Metagenomics Analysis of aMCI and Control Participants

**Figure 3:**
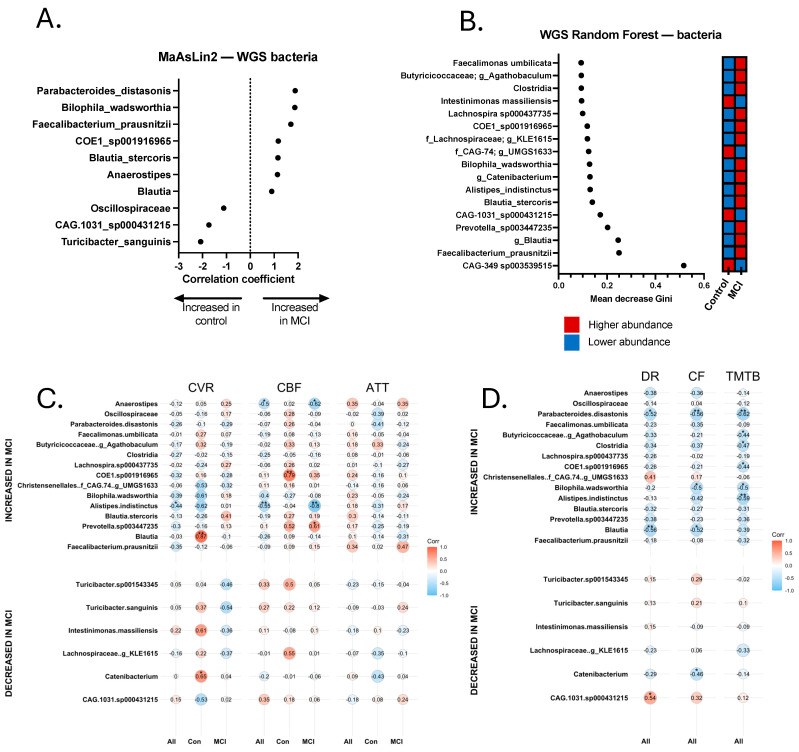
Discriminatory bacteria from metagenomics sequencing Random Forest and MaAsLin2 analyses and correlations with neurovascular and cognitive data. (**A**) Discriminatory taxa identified via MaAsLin2 using a *p*-value cutoff of 0.05. (**B**) Discriminatory taxa identified via Random Forest using a Gini score cutoff of 0.09. Discriminatory taxa underwent Spearman Rho correlational analysis to identify taxa that were significantly correlated with neurovascular metrics, including cerebrovascular reactivity (CVR), cerebral blood flow (CBF), and arterial transit time (ATT). Correlations were calculated separately for all 24 participants (All), 14 aMCI participants (MCI), and 10 controls (Con). Discriminatory taxa also underwent Spearman’s Rho correlational analysis for cognitive analyses, including delayed recall (DR), category fluency (CF), and Trail-Making Test B (TMTB). Correlations for cognitive tests were calculated for the total sample (All). (**C**) Spearman correlations for cerebrovascular analyses. (**D**) Spearman correlations for cognitive tests. * *p* < 0.05, ** *p* < 0.01.

**Figure 4:**
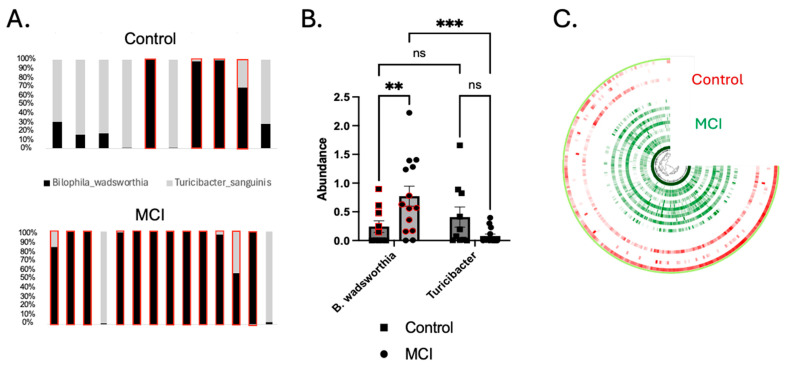
*Turicibacter* was depleted and *Bilophila wadsworthia* was enriched in the aMCI group. (**A**) The ratio of *Turicibacter* to *B. wadsworthia* for individual participants. Each bar plot represents a single participant. Red boxes indicate participants whose concentration of *Bilophila* was greater than that of *Turicibacter.* (**B**) *B. wadsworthia* was significantly enriched in the aMCI group compared to controls (*p* < 0.01), and the ratio of *Bilophila* to *Turicibacter* was significantly higher in the aMCI group (*p* < 0.001) than in controls (*p* > 0.05). *Turicibacter* was enriched in controls, though not significantly so (*p* = 0.093 via the 2-way ANOVA shown above, and *p* = 0.057 via the Mann–Whitney U test comparing only the control vs. the aMCI group). Red circles and squares represent a subset of samples that underwent LEfSe analysis due to similar abundance levels (*p* > 0.9999). (**C**) An anvi’o plot showing the nucleotide variability in the *B. wadsworthia* MAG. The color intensity represents the number of single-nucleotide variants (SNVs) per kb pair. Each ¾ circle represents a single participant (red = control, green = aMCI). ** *p* < 0.01, *** *p* < 0.001.

16S rDNA profiling accounts for taxonomic identification, but cannot accurately identify functionality, which is widely variable between strains of the same species. Thus, we conducted shotgun metagenomics sequencing to identify discriminatory bacterial functions associated with each cohort. After quality controlling the contigs binned with metaBAT2, we ended up with 243 MAGs with an average N50 of 42,341 base pairs (range: 5 kb–389 kb). Many of the MAGs appeared to be the species-level resolution of taxa identified in the 16S dataset (Figure 2). We used both Random Forest (with Boruta) and MaAsLin2 (see Section 2) to identify discriminatory MAGs, which included *Parabacteroides distasonis*, *Bilophila wadsworthia*, *Turicibacter sanguinis*, *Christensenellales CAG-74 UMGS1633*, and *CAG-349* (another member of the *Christensenellales* order). We also observed an enrichment for *Alistipes indistinctus*, *Faecalibacterium prausnitzii*, and *Anaerostipes* in the aMCI group, and an enrichment for Oscillospiraceae, *CAG-1031* (a potential probiotic member of the *Muribaculaceae* family [61] and *Intestimonas massiliensis* in controls. A total of 22 differentially abundant bacterial MAGs were identified (Figure 3A,B), including several with significant and trend-level correlations with the neurovascular and cognitive tests, as detailed below and in Figure 3C,D. *Bilophila wadsworthia*, which has previously been shown to be pro-inflammatory [62], was significantly increased in the aMCI group (*p* < 0.05), whereas *Turicibacter sanguinis*, which has been shown to be negatively correlated with the inflammatory markers IL-1β and IL-6 [63], was enriched in the controls (Figure 4A,B). Another species of *Turicibacter*—*Turicibacter sp001543345*—was enriched in the controls as well. It is worth noting that one control participant was taking fluoxetine at the time of sample collection; fluoxetine is known to deplete *Turicibacter sanguinis* [64], leading us to exclude that sample during analysis of *Turicibacter* abundance. The subsequent analysis of nine controls and 14 aMCI subjects produced statistically significant differences for *Turicibacter* abundance (*Turicibacter sanguinis* and *Turicibacter sp001543345* combined) between the two groups (*p <* 0.05), consistent with the 16S analysis (Figure 2A,B).

Identification of Two Strains of Bilophila wadsworthia. MAGs attributed to *B. wadsworthia* were identified in both the aMCI cohort and controls, but appeared to comprise two strains with extensive single-nucleotide variants (SNVs) across the genome (Figure 4C). To confirm that extensive SNVs corresponded to unique gene composition and functionality, linear discriminant analysis effect size (LEfSe) analysis was conducted on a subset of participants with equal abundances of *B. wadsworthia* (*n* = 5 controls, *n* = 7 aMCI, *p* > 0.999). This analysis yielded 77 unique genes differentially abundant in aMCI and 58 unique genes found in controls (Supplementary File S1). These data support the conclusion that two unique strains of *B. wadsworthia* with unique metabolic properties are present in each cohort.

Relationship between select Bacterial Metagenomes, Neurovascular Patterns, and Cognition. Trend-level and significant correlations emerged between bacterial species and neurovascular and cognitive data (Figure 3C,D). Most notably, *Alistipes indistinctus* was more abundant in aMCI, and was negatively correlated with CVR (*r_s_* = −0.437, *p* = 0.042) and CBF (*r_s_* = −0.546, *p* = 0.007). In addition, *A. indistinctus* was significantly negatively correlated with TMT-B (*r_s_* = −0.587, *p* = 0.005) and category fluency (*r_s_* = −0.422, *p* = 0.050). *B. wadsworthia* sp. was negatively associated (trend-level) with CVR (*r_s_* = −0.389, *p* = 0.074) and CBF (*r_s_* = −0.402, *p* = 0.057), and statistically significantly negatively correlated with cognition, including TMT-A (*r_s_* = −0.444, *p* = 0.044), Trails B (*r_s_* = −0.499, *p* = 0.022), and category fluency (*r_s_* = −0.503, *p* = 0.017). *Anaerostipes* was found to be more abundant in aMCI, and was significantly negatively correlated with CBF (*r_s_* = −0.499, *p* = 0.015), but not with other MRI or cognitive metrics. *Turicibacter* sp. was significantly positively correlated with CBF (*r_s_* = 0.423, *p* = 0.050), but no other significant correlations emerged with neurovascular or cognitive measures. Analyses with *Turicibacter* species excluded the participant who was on fluoxetine at the time of sample collection.

### 3.4. Viral Metagenome Analysis of aMCI and Control Cohorts

**Figure 5:**
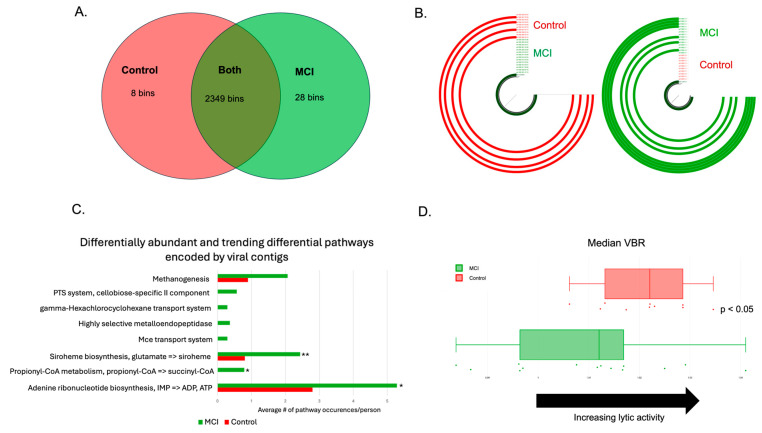
aMCI phageomes were distinct from those of controls. (**A**) Viral bins identified with the control group, the aMCI group, or both after use of a min3 filter. The vast majority of the viral contigs identified were bacteriophages. (**B**) Examples of phage bins identified in (**A**). The bin on the right was only found in the aMCI group (*n* = 7/14, represented by the green ¾ circles), and the bin on the left was only found in the controls (*n* = 4/10, represented by the red ¾ circles). (**C**) Differential metabolic pathways encoded by phage contigs in the aMCI group vs. the control group. Statistically significant comparisons are denoted by the following: *p* < 0.05: *; *p* < 0.01: **. (**D**) Assessment of phage lifestyle using the viral-to-bacterial ratio (VBR). Values to the right indicate an increase in lytic activity, while values to the left indicate lysogeny. Controls (red dots and boxplot) are more likely to have a more lytic VBR, whereas aMCI participants (green dots and boxplot) have a more lysogenic gut virome (*p* < 0.05).

The human gut harbors between 10^8^ and 10^10^ viral-like particles per gram of feces [65]. Thus, the shotgun metagenomics sequencing data were also analyzed using standard tools (see Section 2) for differentially abundant viruses. We identified 2387 high-quality viral contigs, the vast majority of which were identified to be bacteriophages. LEfSe analysis revealed that 36 phage contigs were differentially abundant between the cohorts, while MaAsLin2 revealed 74 differentially abundant phage contigs. Thirty phage contigs were identified by both methods. An additional nine contigs that were identified via LEfSe had trend-level significance (*p* < 0.10) in MaAsLin2 to differentiate the cohorts. Furthermore, there were 8 phage contigs only present in the control cohort, and 28 phage contigs only present in aMCI participants (Figure 5A). The data were used to predict phage-encoded metabolic pathways, identified using DRAMv. Multiple metabolic functions were found to be differentially abundant between the two cohorts, including ATP and ADP synthesis from inosine monophosphate, propionyl-CoA metabolism, siroheme biosynthesis, and methanogenesis (Figure 5C). Lastly, because phages display specific lifestyles, including lysis and lysogeny, we assessed the viral-to-bacterial ratio (VBR) to predict viral lifestyle, as described previously (see methods) [52]. We found that viruses associated with aMCI were significantly more likely to be lysogenic than lytic, compared to the controls (Figure 5D, *p* < 0.05).

**Figure 6:**
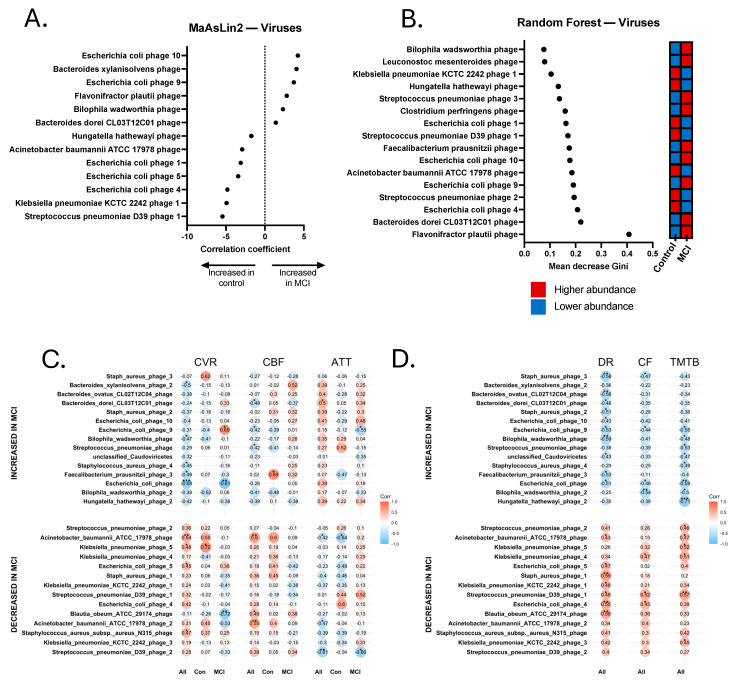
Discriminatory viruses from metagenomics sequencing Random Forest and MaAsLin2 analyses and correlations with neurovascular and cognitive data. (**A**) Discriminatory viral contigs identified via MaAsLin2 using a *p*-value cutoff of 0.05. (**B**) Discriminatory viral contigs identified via Random Forest using a Gini score cutoff of 0.09. Discriminatory viral contigs underwent Spearman Rho correlational analysis to identify contigs that were significantly correlated with neurovascular metrics, including cerebrovascular reactivity (CVR), cerebral blood flow (CBF), and arterial transit time (ATT). Correlations were calculated separately for all 24 participants (All), 14 aMCI participants (MCI), and 10 controls (Con). Discriminatory viral contigs also underwent Spearman’s Rho correlational analysis for cognitive tests, including delayed recall (DR), category fluency (CF), and Trail-Making Test B (TMTB). Correlations for cognitive tests were calculated for the total sample (All). (**C**) Spearman correlations for cerebrovascular analyses. (**D**) Spearman correlations for cognitive tests. * *p* < 0.05, ** *p* < 0.01, *** *p* < 0.001.

Relationship between Select Viral Contigs, Neurovascular Patterns, and Cognition. Finally, viral contig abundance was significantly correlated with the neurovascular and cognitive data. We found that several phage contigs were significantly associated with clinical and/or neurovascular measures, including phages that are associated with *Bacteroides ovatus*, *Roseburia intestinalis*, and *E. coli* (Figure 6). Significant positive and negative correlations were revealed between several phage contigs, CVR, CBF, ATT, TMT A and B, delayed recall, and category fluency. It is worth noting that one *Bilophila wadsworthia* phage was identified as enriched in aMCI, and was significantly negatively associated with delayed recall (*r_s_* = −0.589, *p* = 0.004) and TMT-B (*r_s_* = −0.491, *p* = 0.033). This phage was also significantly negatively associated with CVR (*r_s_* = −0.474, *p* = 0.026), had a trend-level positive association with ATT (*r_s_* = 0.352, *p* = 0.099), and contained an acyl-CoA synthetase capable of influencing central metabolism (Figure 6). Additionally, a phage associated with *Acinetobacter baumannii ATCC 17978* that was enriched in controls was significantly positively associated with nearly all tested metrics, including CVR (*r_s_* = 0.543, *p* = 0.009), CBF (*r_s_* = 0.599, *p* = 0.0025), delayed recall (*r_s_* = 0.427, *p* = 0.048), and TMT-B (*r_s_* = 0.467, *p* = 0.033), and was significantly negatively associated with ATT (*r_s_* = −0.424, *p* = 0.044).

## 4. Discussion

After decades of research, Alzheimer’s disease remains a devastating and costly neurodegenerative condition without an effective treatment. Increasingly, there is promising evidence that the GMB may change our understanding of AD at all phases of the disease, which includes the preclinical, prodromal (MCI), and dementia stages. Comprehensive investigation of the gut–brain axis throughout AD progression is urgently needed to elucidate both direct and indirect mechanisms by which gut microbial communities may influence central nervous system function, either promoting or mitigating AD progression. Neurovascular changes can occur decades before the onset of AD symptomatology, and disruption of neurovascular structures allows the influx of pathogens into the brain [14,15,16]. However, while there have been a couple of studies examining associations between the GMB and neurovascular and cognitive function in mice [19,66], there is no known human study that has yet examined connections between the GMB and neurovascular changes in prodromal AD (aMCI due to AD). Therefore, we evaluated the associations between the GMB, neurovascular functioning as measured by MRI, and cognition in aMCI and control cohorts. To the best of our knowledge, this is the first study that examines the relationship between the GMB, including both bacteria and viruses, and neurovascular changes in aMCI.

Consistently with previous studies examining neurovascular changes in AD (e.g., ref. [24]), we found that aMCI participants had lower CVR and CBF and a longer ATT compared to the control group (Table 2 and Figure 1), and that CVR and CBF were associated with cognition (Table 3). Similarly to previously published results [67], 16S sequencing analysis revealed an increase in pro-inflammatory bacterial taxa in the aMCI cohort, such as Bilophila and Erysipelotrichaceae UCG-003, and an enrichment of probiotic ASVs in the controls, including Akkermansia, Subdoligranulum, and Turicibacter. We found that several of the enriched ASVs for each cohort had many significant (*p* < 0.05) and trend-level (*p* < 0.10) correlations with the cognitive and neurovascular data. More specifically, ASVs enriched in aMCI had negative correlations with CVR and CBF and positive correlations with ATT. The opposite trend was true for ASVs enriched in controls, for which abundance was positively correlated with CVR and CBF and negatively correlated with ATT. Further, inflammation has been implicated in neurovascular changes associated with AD [68], suggesting a possible biological relationship between bacterial presence and neurovascular function.

Since 16S sequencing does not generally provide species- or strain-level resolution, nor does it provide functional information, we turned to shotgun metagenomics sequencing to identify specific bacterial species and their associated metabolic pathways that might be enriched in our two cohorts. Similarly to the 16S data, we identified several pro-inflammatory bacterial metagenomes enriched in the aMCI group and several health-promoting MAGs enriched in the controls. Notably, we saw an enrichment for Alistipes indistinctus in the aMCI group, as well as significant correlations between this bacterium and measures of both neurovascular and cognitive function. Interestingly, A. indistinctus has been shown to deplete intestinal urate levels [69], which is a risk factor for AD [70]. Bilophila wadsworthia was also enriched in the aMCI group, and significantly correlated with multiple cognitive metrics. B. wadsworthia has been shown to deplete host urate levels via the breakdown of taurine (from bile acids) into hydrogen sulfide, which interferes with the production of urate [71]. In contrast, Turicibacter, which was enriched in controls and is associated with an increase in intestinal taurine levels, has a positive correlation with intestinal urate levels [71]. Further, both Bilophila and Turicibacter are directly implicated in bile acid metabolism, and altered bile acid profiles have been associated with neurodegeneration, including in MCI and AD [72]. For instance, Nho et al. [72] demonstrated bile acid signatures that were associated with CSF Aβ1-42, *p*-tau 181, t-tau, glucose metabolism, and atrophy by combining neuroimaging techniques with targeted metabolomics. We did not see statistically significant correlations between neurovascular patterns and Bilophila and Turicibacter levels using our metagenomics data, despite there being a significant association between Bilophila and CVR and between Turicibacter and CBF in our 16S data. The reason for this discrepancy could be due to loss of lower-quality Bilophila and Turicibacter sequences, resulting in the formation of MAGs that did not fully represent those bacterial taxa. Both Turicibacter MAGs were less than 70% complete; thus, if those MAGs were complete, there may have been less divergence between the 16S and shotgun sequencing data. However, trend-level associations between Bilophila and CVR and between Turicibacter and CBF were still identified, indicating that significance may be reached with a larger sample size than we had available for this study. Additionally, Bilophila was significantly associated with TMT A and B, as well as category fluency, further demonstrating its relationship to cognitive functioning. Several of the other differentially abundant bacterial MAGs that were enriched in the aMCI group seem to have conflicting findings within the current literature. For example, Vogt et al. [4] saw similar trends to us, whereby Blautia and Alistipes were associated with cognitive impairment. Li et al. [73] also saw an increase in Blautia in MCI, but unlike us, they observed a decrease in Alistipes and Parabacteroides. Further, Liu et al. [9] observed a decrease in Blautia in aMCI patients. There are several possible reasons for the discrepancies between all of these studies. For one, there are cultural and environmental differences in patient populations (e.g., participants from China vs. the United States), leading to potential differences in GMB results. Another potential explanation is differences in the identified bacterial strains. There are several species within each genus of bacteria and several strains within each species; thus, while one Blautia species may be pro-inflammatory, another may be anti-inflammatory or neutral, depending on the differences in metabolism between the species or strains. Another example of this is Parabacteroides distasonis, which was increased in our aMCI cohort. In one study, *P. distasonis* was positively associated with post-operative delirium [74], and in another, it had a negative association with neurocognitive development [75]. However, it seems that *P. distasonis* has an ambivalent association with inflammation, as it has been shown to be both probiotic and anti-inflammatory [76], depending on the context.

Finally, we assessed differences in the gut virome between controls and aMCI individuals. It has been demonstrated that previous viral infections with known pathogens, such as HSV-1, influenza, SARS-CoV-2, and viral encephalitis, increase the risk of developing AD [77,78,79]. Previous work has shown that HSV-1 infection directly modulates tau expression and pathologies [80,81], and that SARS-CoV-2 can infect the CNS and contribute to neuroinflammation [82], suggesting that viruses may be directly capable of causing neuronal damage and contribute to AD progression. However, not all types of viruses have been examined in the context of neurodegeneration. Bacteriophages, or phages, which are thought of as inherently non-pathogenic, are highly prevalent in the gut, with feces containing about 10 [9] viral-like particles per gram [83]. While phages are not thought to directly cause disease, several research groups have identified connections between the gut virome (which primarily consists of phages) and disease [84,85,86], possibly through altered interactions with the host bacterial population and/or the immune system. So far, one study has examined non-pathogenic gut viruses as possible contributors to AD-related cognitive decline as assessed with a cognitive screening instrument [11], but no studies have examined the relationship between gut viruses, sensitive neuropsychological tests, and neuroimaging. We identified differentially abundant phages, which may have the ability to metabolically reprogram their host bacterial physiology, thereby shifting the overall metabolic profile in the gut [87]. Phage-associated metabolic pathways are known to contribute to secondary metabolite production and bile acid metabolism [88,89], ultimately changing the circulating metabolite profile within the host. Phages associated with aMCI were also more likely to be capable of lysogeny (integrating into the bacterial genome; Figure 5D, *p* < 0.05), allowing for stable transmission within bacterial cells over time. Bacteria-phage stability could affect both protective or pathogenic features in either control or MCI cohorts. However, Johansen et al. [90] demonstrated that the gut virome (phageome) trends towards a more lytic state as people age. Lytic phages lead to a decrease in bacterial populations overall, conferring an element of passive immunity. Thus, our results suggest that the increase in lysogenic phages in the aMCI cohort represents a disruption in the normal aging GMB, and may represent a loss of passive immunity to specific pathogenic bacteria capable of influencing neurodegeneration. Overall, our results suggest that disruption of the gut microbiota, including phage dynamics, alters the metabolic functionality of the gut ecosystem in aMCI individuals relative to controls. Metabolic features of the microbiota may affect pro-inflammatory metabolites and circulating bile acids that, when combined with age-related BBB permeability changes, lead to accelerated neurovascular changes and neurodegeneration.

However, it is important to note the limitations in this study. For one, due to the cohort composition, we were unable to determine a potential role of race and ethnicity, medical history, and environmental factors in the aMCI-associated GMB and associated neurovascular dysfunction. We also acknowledge the small sample size used for this study which may also have contributed to the number of trend-level, rather than significant, correlations identified between the gut microbiota, neurovascular dysfunction, and cognition. Since this is a highly novel proof-of-concept study that has limited statistical power, we prioritized minimizing Type II error to avoid overlooking meaningful associations that emerged in our Spearman correlational analyses. We reported bacterial taxa and phages that showed at least one statistically significant or trending correlational value with neurovascular and/or cognitive measures. This approach is consistent with our goal of identifying preliminary microbial signals that may warrant further investigation in larger, hypothesis-driven studies. We plan to further validate our results in our future work with larger sample sizes. We are repeating this study with additional participants to further validate the results shown here, along with the addition of other critical data elements (e.g., bacterial metabolites). We intend to conduct additional statistical analyses (e.g., multivariate linear regression to model bacterial and viral composition predictors of neurovascular functioning) and examine the influence of covariates such as pre-existing cardiovascular conditions. It will also be important to conduct a similar study, but in a longitudinal manner, to assess changes to the GMB and neurovascular function over time. Further, we plan to assess blood (plasma) for markers of vascular function and AD biomarkers, including Aβ and *p*-tau, and determine whether significant associations exist between these biomarkers, neurovascular changes using MRI, and dysbiosis. Lastly, we plan to obtain metabolomics data for aMCI and control cohorts, including quantifying the production of SCFAs and bile acids. Through active collaborations with communities and academic institutional partners to conduct community-engaged participatory research, we will work to improve research equity in our future gut–brain axis scientific endeavors. Future studies will likely include a rodent model to test whether specific bacteria, phages, or metabolites can drive neurodegeneration in vivo. This study provides a connection between gut dysbiosis, cognitive functioning, and neurovascular changes associated with aMCI, paving the way for future studies to identify novel biomarker testing and therapeutic targets for neurodegeneration, cognitive decline, and AD.

## 5. Conclusions

Herein, we examined the interplay between the gut microbiome, cognitive decline, and neurovascular function. We examined differences in gut microbiome composition in patients diagnosed with prodromal AD/aMCI as compared to cognitively unimpaired older adult controls. We then found associations between microbiome differences and cognitive and neurovascular functioning using Spearman’s Rho correlational analyses. We found that several bacteria associated with inflammation were enriched in the aMCI group, and generally had negative correlations with delayed recall, category fluency, TMT-B, CVR, and CBF, and positive correlations with ATT. Conversely, bacteria enriched in the controls generally had positive correlations with all of these measures of cognitive and neurovascular function and negative correlations with ATT. We also found that controls and aMCI individuals had different bacteriophage populations, with the aMCI phage population more likely to be capable of lysogeny, and were enriched for different biochemical pathways compared to the control phage population. Notably, the phages enriched in the aMCI group were negatively correlated with cognition, CBF, and CVR, and positively correlated with ATT, whereas the reverse was true for phages enriched in controls. Overall, our results are consistent with the growing body of literature showing that neurodegeneration is associated with an altered microbiome, and that neurovascular changes are detectable even at the prodromal stage of AD. Further, this study is the first known human study to provide a connection between the microbiome and neurovascular function, and we hope that it will act as a stepping stone for future work to validate and identify molecular mechanisms for these connections.

## Figures and Tables

**Table 1:** Sample characteristics for the amnestic MCI (aMCI) and control groups.

	aMCI	Controls	t/χ^2^	*p*
*n*	14	10		
Age (M ± SD)	73.21 ± 6.14	70.70 ± 6.13	−0.990	0.333
Education (M ± SD)	15.00 ± 2.00	16.80 ± 2.97	1.78	0.089
Sex	7 F/7 M	7 F/3 M	0.960	0.421
BMI	26.68 ± 4.76	26.06 ± 4.33	−0.324	0.749
Mother with dementia	7 no/7 yes	9 no/1 yes	4.20	0.079
Father with dementia	13 no/1 yes	6 no/4 yes	3.82	0.122

Note. BMI = body mass index. t = t statistic from independent-sample *t*-tests for age, education, and BMI. χ^2^ = chi-square test results for sex and parental history of dementia.

**Table 2:** Means, SDs, and group comparisons for cognitive measures and MRI data (CVR, CBF, ATT) for control group vs. amnestic MCI (aMCI) group.

Measure	Controls M (SD)	*n*	aMCI M (SD)	*n*	t/*z*	*p*
TMT A	114.80 (13.35)	10	95.36 (17.15)	11	2.88	0.005
TMT B	117.80 (14.26)	10	89.00 (20.79)	11	3.66	0.001
Letter fluency	102.40 (15.61)	10	99.42 (9.56)	12	0.55	0.294
Semantic fluency	110.90 (12.58)	10	87.67 (15.89)	12	3.74	<0.001
Delayed recall	111.50 (14.15)	10	69.33 (11.36)	12	7.76	<0.001
CVR	1.01 (0.36)	10	0.59 (0.21)	12	−2.84	0.003
CBF	52.05 (12.09)	10	36.91 (9.59)	13	−2.54	0.010
ATT	1442.74 (110.47)	10	1629.38 (169.89)	13	2.48	0.012

Note: Independent *t*-tests were performed for cognitive measures. Mann–Whitney’s *U* results are reported for CVR, CBF, and ATT group comparisons. Values for cognitive measures are standard scores (M = 100, SD = 15). TMT = Trail-Making Test. Delayed recall = spontaneous free recall after long delay on a word list memory measure. CVR = cerebrovascular reactivity, CBF = cerebral blood flow, and ATT = arterial transit time.

**Table 3:** Spearman correlations for cognitive and neurovascular measures.

	CVR		CBF		ATT	
	*r_s_*	*p*	*r_s_*	*p*	*r_s_*	*p*
TMT A	0.283	0.240	0.315	0.176	−0.148	0.534
TMT B	0.527	0.020	0.417	0.067	−0.357	0.122
Sem. fl.	0.257	0.275	0.512	0.018	−0.332	0.141
Memory	0.432	0.057	0.376	0.093	−0.365	0.104

Note. CVR = cerebrovascular reactivity, CBF = cerebral blood flow, and ATT = arterial transit time. TMT = Trail-Making Test. Sem. fl. = semantic fluency. Memory = spontaneous free recall after long delay on a word list memory measure.

## Data Availability

The data supporting the findings of this study are available on request from the corresponding author. This is an ongoing study. The data are not publicly available due to privacy or ethical restrictions. All DNA sequencing data generated from these studies have been made available in the National Library of Medicine, National Center for Biotechnology Information, Sequence Read Archive as BioProject ID PRJNA1161622.

## Supplementary Materials

The following supporting information can be downloaded at: https://www.mdpi.com/article/10.3390/brainsci15060538/s1, File S1: *Bilophila wadsworthia* subset analysis.xlsx
